# Multiple stressors disrupt sex hormones and fitness outcomes: effects of hypoxia and turbidity on an African cichlid fish

**DOI:** 10.1093/conphys/coae066

**Published:** 2024-10-22

**Authors:** Bethany L Williams, Lauren M Pintor, Jai Tiarks, Suzanne M Gray

**Affiliations:** School of Environment and Natural Resources, 2021 Coffey Rd, The Ohio State University, Columbus, OH 43210, USA; Department of Biology, University of Missouri–St. Louis, 1 University Blvd, St. Louis, MO 63121, USA; School of Environment and Natural Resources, 2021 Coffey Rd, The Ohio State University, Columbus, OH 43210, USA; School of Environment and Natural Resources, 2021 Coffey Rd, The Ohio State University, Columbus, OH 43210, USA; School of Environment and Natural Resources, 2021 Coffey Rd, The Ohio State University, Columbus, OH 43210, USA; Department of Biology, University of Prince Edward Island, 550 University Ave, Charlottetown, PE, C1A 4P3 Canada

**Keywords:** Aromatase, cichlid, fish, hypoxia, testosterone, turbidity

## Abstract

Freshwater organisms face a complex array of environmental stressors that can negatively affect endocrine function and subsequent fitness outcomes. Hypoxia and turbidity are two environmental stressors that are increasing due to human activities that could lead to endocrine disruption and reduced reproductive output. Our research addresses how hypoxia and elevated turbidity affect traits related to reproductive success, specifically sex hormone concentrations, investment in reproductive tissues and body size. We used wild fish from two populations (a river and a swamp) of an African cichlid, *Pseudocrenilabrus multicolor*, to produce offspring that were reared in a full factorial split brood rearing experiment (hypoxic/normoxic × clear/turbid). River and swamp populations represent divergent habitat types with respect to the stressors of interest, being well-oxygenated but turbid or hypoxic and clear, respectively. Overall, we found evidence for plastic responses to both stressors. Specifically, we found that there was an interactive effect of oxygen and turbidity on testosterone in males from both populations. Additionally, males of both populations reared under hypoxic conditions were significantly smaller in both mass and standard length than those raised under normoxic conditions and invested less in reproductive tissues (quantified as gonadosomatic index). Hypoxia and turbidity are experienced naturally by this species, and these environmental stressors did not affect the number of eggs laid by females when experienced in the absence of another stressor (i.e. normoxic/turbid or hypoxic/clear). However, there was an interactive effect of hypoxia and turbidity, as females reared and maintained under this treatment combination laid fewer eggs. This research underscores the importance of considering the possibility of stressor interactions when determining how anthropogenic stressors affect fitness outcomes.

## Introduction

Freshwater organisms face a complex array of environmental stressors that can negatively affect endocrine function and subsequent fitness outcomes. Threats facing freshwater ecosystems that cause or elevate stressors include habitat degradation, climate change, harmful algal blooms, pollution and invasive species (Reid *et al.,* 2019). In particular, endocrine disruption caused by habitat degradation (e.g. pollutants and hypoxia) can sharply decrease fitness because sex hormones are responsible for stimulating gametogenesis, inducing spermiation, controlling sexual behaviour and regulating secondary sexual characteristics (Wu, 2009; Munakata and Kobayashi, 2010; Söffker and Tyler, 2012). The endocrine system is essential for an organism’s ability to respond to changes in its environment (Duffy *et al.,* 2002). However, hormone production by the endocrine system is also directly affected by environmental stressors, impacting an organism’s reproductive behaviour and fitness (Pankhurst and Munday, 2011). In addition to disrupting endocrine function, environmental stressors can affect other key characteristics that determine fitness outcomes, such as body size (Kingsolver and Pfennig, 2004, 2007) and energy available for reproduction (Landry *et al.,* 2007).

The environment can affect hormone synthesis directly (e.g. temperature affecting rates of hormone synthesis; Pankhurst and Munday, 2011) and indirectly (e.g. hypoxia reducing energy available to invest in reproduction; Landry *et al.,* 2007). Examples of environmental stressors that can alter hormone production include increased temperature, hypoxia and pollutants (Wu *et al.,* 2003; Landry *et al.,* 2007; Martinovíc *et al.,* 2007; Candolin and Wong, 2019). For example, higher temperatures increase the reaction rate of hormone synthesis in ectothermic animals, and at temperatures above the thermal tolerance range, hormone synthesis can be inhibited due to changes in protein structure that impair their function (Pankhurst and Munday, 2011). Pollutants like synthetic oestrogens can skew sex ratios towards females (Candolin and Wong, 2019) and lead to lower levels of androgens in males (Martinovíc *et al.,* 2007). Furthermore, the environment can affect reproductive hormones indirectly via chronic activation of the stress axis, which can inhibit reproduction (Tilbrook *et al.,* 2000).

In addition to affecting hormone production, environmental stressors are also likely to affect other traits related to reproductive success such as body size. Fitness is directly related to body size in various invertebrates, vertebrates and plants (Kingsolver and Pfennig, 2004). Larger body size is often correlated with higher survival, greater fecundity and increased mating success (Kingsolver and Pfennig, 2004; Kingsolver and Pfennig, 2007). Larger males are more likely to hold higher quality territories, have greater access to females and engage in more courtship behaviours (Johnson and Hixon, 2011). In female teleost fish, fecundity is exponentially scaled with body size (Hixon *et al.,* 2014; Barneche *et al.,* 2018). However, body size can also be influenced by environmental factors such as increasing temperature, hypoxia, size-selective fisheries, food availability and predation (Gardner *et al.,* 2011; Ahti *et al.,* 2020). For example, in ectotherms, temperature can affect body size directly by altering growth and development rates or indirectly by altering species interactions and food availability (Ohlberger, 2013)*.* Fish that develop under hypoxic conditions also tend to be smaller than fish reared under normoxic conditions (Chapman, 2021). These examples explore the effect of single environmental stressors; however, in natural systems, we expect organisms to be faced with a suite of stressors, especially in highly degraded habitats.

Multiple stressors can affect traits independently in a linear fashion (i.e. additively). Alternatively, responses to stressors can be identified as antagonistic (a non-linear interaction that is lesser than the sum of the individual stressors) or synergistic (a non-linear interaction that is greater than the sum of the individual stressors) (Folt *et al.,* 1999; Townsend *et al.,* 2008; Todgham and Stillman, 2013; Piggott *et al.,* 2015; Côte *et al.,* 2016). Hypoxia and turbidity are examples of two environmental stressors increasing in aquatic systems globally due to human activities that could interact to affect fitness (Diaz and Rosenberg, 2008; Gray, 2016; Jenny *et al.,* 2016). Hypoxia is commonly defined as dissolved oxygen (DO) levels below 2.0 mg O_2_/l, though species vary in their tolerance to hypoxia (Farrell and Richards, 2009). In addition to lethal effects, hypoxia directly disrupts endocrine function because it inhibits the aromatase enzyme, an enzyme found in the gonads and brain that converts testosterone to oestradiol (Thomas *et al.,* 2007; Huffman *et al.,* 2013). Hypoxia can generally reduce the energy available for reproduction and hormone production (Wu *et al.,* 2003; Landry *et al.,* 2007; Friesen *et al.,* 2012; Cheung *et al.,* 2014). Turbidity (i.e. suspended particulate matter) is also increasing in many aquatic ecosystems due to human activities like deforestation, which increases the runoff of sediments into aquatic ecosystems (Gray, 2016). In turbid water, light availability is reduced, and the colour of light may be altered (Gray, 2016). Such changes to the visual environment can lead to changes in important reproductive behaviours. For example, male guppies (*Poecilia reticulata*) reared and tested in turbid environments perform fewer courtship displays than those reared and tested in clear water (Camargo-dos-Santos *et al.,* 2021). Additionally, turbid conditions alter mate choice decisions in sticklebacks (*Gasterosteus aculeatus*), which primarily use visual cues in clear conditions and olfactory cues in turbidity conditions (Heuschele *et al.,* 2009). Turbid environments can also affect aquatic organisms directly by damaging respiratory tissues or smothering eggs (Gray *et al.,* 2012a; Gray, 2016). Overall, hypoxia and turbidity influence several traits important for reproduction, but their potential combined effect on reproduction is less understood. However, since both stressors affect multiple traits that are related to reproduction, experiencing both hypoxia and turbidity simultaneously could have an additive or synergistic effect on reproductive outcomes.

Anthropogenic changes in oxygen and turbidity could have profound effects on reproductive success by altering sex hormone production, body size or energy available to invest in reproduction. *Pseudocrenilabrus multicolor* is a widespread African cichlid found in swamps, lakes and rivers across the Nile River drainage and Lake Victoria basin (Reardon and Chapman, 2009; Gray *et al.,* 2012b). Reproduction in *P. multicolor* is not confined to a single breeding season and occurs multiple times throughout the year (Reardon and Chapman, 2008). Males will first dig (and defend) a territorial pit, to which they attract females by engaging in lateral displays (dorsal and anal fins completely erect and fish perpendicular to female) and quivers (rapid shaking motion of the entire body; Gray *et al.,* 2012b). It is unclear whether fertilization of the eggs occurs on the substrate or in the females’ mouth (Reardon and Chapman, 2010), but after fertilization, females mouthbrood for approximately 2–3 weeks and then release their offspring as juveniles (Reardon and Chapman, 2012). Maternal mouthbrooding is a costly form of parental care as females cannot eat while brooding (Reardon and Chapman, 2009). Conditions in swamps where *P. multicolor* is found tend to be consistently hypoxic and clear (although tannin-stained), while river sites are generally normoxic and variably turbid (Gray *et al.,* 2012b). Aquatic habitats in the Lake Victoria region are also subject to several human activities that alter environmental conditions such as deforestation, eutrophication and the introduction of non-native species (Chapman *et al.,* 2008). Previous work indicates that hypoxia induces life history trade-offs in *P. multicolor*, including the production of a larger number of smaller eggs, slower growth and smaller size at maturity (Reardon and Chapman, 2009; Chapman, 2021). Additionally, females from swamp sites experience aromatase inhibition (Friesen *et al.,* 2012), which can lead to ovarian masculinization and impaired gamete production in other species of fish (Thomas *et al.,* 2007). Overall, *P. multicolor* readily survives at these extremes of oxygen and turbidity concentrations, making it an excellent model species to study the independent and interactive effects of oxygen and turbidity on sex hormones, body size and reproductive investment.

Our goal was to assess the relative effects of the population (swamp or river) and multiple stressors (hypoxia and turbidity) on sex hormone concentrations in males, investment in reproduction in both males and females [quantified as gonadosomatic index (GSI) in both males and females] and egg count in brooding females. Additionally, we examined morphological traits [standard length (SL) and mass] in males and females, as these size metrics are important determinants of fitness in fish. Because a previous study has examined the effect of hypoxia on hormones in female *P. multicolor* (Friesen *et al.,* 2012), we chose to focus on the effects of hypoxia and turbidity only in males. Based on this previous study (Friesen *et al.,* 2012), we predicted that hypoxia would increase the ratio of testosterone to oestradiol (indicative of aromatase inhibition) in males. Furthermore, based on behavioural responses to turbidity (increased aggression in male competition trials; Gray *et al.,* 2012b), we predicted that turbidity would increase testosterone levels, which is associated with higher territoriality and dominance (Parikh *et al.,* 2006)*.* To test these questions, we measured sex hormones in males, GSI, egg count and morphological measurements in F1 fish from a full factorial split brood rearing experiment using fish from one swamp and one river population. We predicted that hypoxia and turbidity would interact to negatively affect reproduction due to the combination of changes in energy availability, hormones and female body size that we expected to see when fish were reared under the hypoxic and turbid treatment combination.

## Materials and Methods

### Ethics statement

Research was conducted under approval from The Ohio State University Institutional Animal Care and Use Committee (2014A00000055-R1). Scientific permits were obtained from the Commissioner of Fisheries Resources Management and Development, Uganda, for permission to export fish and from the Uganda National Council for Science and Technology for research permission.

### Rearing experiment

To assess the effects of hypoxia and turbidity on hormone levels and fitness metrics, we measured testosterone and oestradiol in F1 *P. multicolor* males derived from wild-caught fish reared in a split brood rearing experiment (full factorial, hypoxic/normoxic × clear/turbid treatments). Fish used as parents in this rearing study were collected from one swamp site (Lwamunda) and one river site (Ndyabusole) in the Lake Nabugabo region of Uganda in May 2018. The swamp site is typically hypoxic (0.79 ± 0.1 mg/l, mean ± SE, point in time measurements) and clear, albeit tannin-stained [2.02 ± 0.3 nephelometric turbidity units (NTU)], while the river site is typically normoxic (6.84 ± 0.2 mg/l) and moderately turbid (18.81 ± 1.1 NTU); measurements were taken between June and August in 2015, 2016 and 2017 from Oldham (2018). Ten broods from each of two populations (one river and one swamp population) were reared under each combination of rearing conditions. Once a female released her brood, the young were housed for 1 week under normoxic, clear conditions before being split randomly into the four different treatment groups. In the hypoxic treatment group, the oxygen was reduced gradually over a period of 1 week by placing bubble wrap over the surface of the water. In the turbid tanks, turbidity was gradually increased over a 1 week period by adding ~1.5 ml of bentonite clay solution (100 g clay/l water). DO concentrations were measured throughout the rearing experiment (DO: hypoxic mean ± SE = 2.27 ± 0.01 mg/l O_2_ or normoxic mean = 7.52 ± 0.001 mg/l O_2_) using a YSI Pro2030 multimeter probe 3–7 days per week and was adjusted as needed by adjusting bubble wrap and bubbling water with ambient air or nitrogen gas. Turbidity (clear: 1.31 ± 0.03 NTU or turbid: 16.6 ± 0.14 NTU) was measured 1–2 days per week using a Hach2100Q portable turbidimeter and was adjusted as needed during weekly water changes or through the addition of the bentonite clay solution. Fish were fed TetraMin Tropical crisps once a day *ad libitum* for 5 minutes. The treatments and populations were dispersed randomly across 80 aquaria to minimize small differences in light and temperature (mean ± SE: 24.9 ± 0.02°C) (Williams, 2023). We maintained fish in a 12L:12D photoperiod. All fish were mature at the time of sampling. To distinguish between fish within a tank, we used white visible implant elastomer tags. We tagged 348 fish (156 males and 192 females) when they were between 345 and 386 days old.

The results reported here are part of a larger study where male and female reproductive behaviours (Williams *et al.,* 2024) were examined using the fish described here. For additional details of the rearing experiment components, see Williams (2023), Williams *et al.* (2024) and Tiarks (2024). We used subsets for measures of hormones (*n* = 77, when males were between 466 and 514 days old) and egg count (*n* = 37, when females were between 615 and 794 days old). After hormone and fecundity and reproductive behaviour (Williams *et al.,* 2024) data were collected, we sampled fish for morphological metrics (*n* = 255) and GSI (*n* = 234). The age of fish ranged from 571 to 827 at the time of final sampling due to restrictions to laboratory access during the COVID-19 pandemic.

### Hormone collection

Hormone samples were collected from a subset of adult males between 24 June 2020 and 5 July 2020, when fish (*n* = 77) were between 466 and 514 days old (fish were maintained at treatment conditions from 7 days postrelease). Hormones were collected between 09:30 and 13:30 to minimize diel variation in hormone concentrations (Cowan *et al.,* 2017). We collected hormone samples using a non-invasive method that has been previously validated in *P. multicolor* and other species of fish (Kidd *et al.,* 2010; Friesen *et al.,* 2012). Hormone samples were collected by placing individual fish in glass containers containing 200 ml of clean treated water for 1 h, during which time hormones are released from the gills at a rate that is highly correlated with the concentration in plasma (Friesen *et al.,* 2012). The oxygen and turbidity of the sampling water matched the rearing treatment the fish was raised in. Hormone samples were extracted onto Sep-Pak Plus C18 SPE cartridges and frozen at −20°C until further processing. Hormones were extracted from the cartridges using ethyl acetate and dried under a stream of nitrogen gas. Samples were then reconstituted in assay kit buffer (582 701 and 582 251, Cayman Chemical) according to manufacturer instructions. To quantify background levels of hormones in the water, four water samples not containing fish were also collected, and the concentrations of testosterone and oestradiol were measured to establish a baseline level. The average baseline measurement of hormones (mean ± SE = 85.3 ± 12.7 pg/ml testosterone and 119.0 ± 17.0 pg/ml oestradiol) was subtracted from the hormone measurements. Sample measurements lower than the baseline water measurements were replaced with zeroes for analysis (5 testosterone, 38 oestradiol measurements). Because oestradiol measurements were below the detection threshold for many individuals, we did not statistically analyse oestradiol by itself, only the ratio of testosterone/oestradiol. Oestradiol averages are presented in Supplementary Fig. S1.

### Morphological measurements

SL (cm; length from tip of snout to end of caudal peduncle) and mass (g) were collected before euthanizing (in 1:10 eugenol:ethanol solution) males (*n* = 118) and females (*n* = 137; see Supplementary Table S1 for treatment sample sizes) at the end of the rearing experiment.

### Fitness metrics

To understand the effects of the rearing environment and population on investment in reproduction (Brewer *et al.,* 2008), we analysed GSI in a subset of males (*n* = 107) and females (*n* = 127). The gonads were removed, and we calculated GSI using the following formula: GSI = (gonad mass/mass) ^*^ 100.

To quantify the effects of the rearing treatments on fitness more directly, we placed a subset of sexually mature females from the rearing experiment in aquaria with non-sibling males of the same rearing treatment and population group. The males and females were not used as parents more than one time, so each brood has a distinct combination of parents. Rearing treatment conditions were maintained in these aquaria. Females were observed daily, and when they were determined to be carrying a brood, we removed the brood from her mouth, counted the number of eggs and weighed the brood (ME104E, Mettler Toledo analytical balance) to determine brood mass (g; *n* = 37 broods; two to six broods per treatment/population combination). We then calculated the average egg mass by dividing the brood mass by the number of eggs. Average egg mass and number of eggs in the brood were used as proxies for reproductive success.

### Statistics

All analyses were conducted in R, version 4.3.0 (R Core Team, 2024). Linear mixed models (LMMs) were performed using *Lme4*, version 1.10–35.1 (Bates *et al.,* 2015). To understand the interacting and independent effects of oxygen, turbidity and population on sex hormones and fitness metrics, all models contained oxygen, turbidity, the interaction between oxygen and turbidity, and population as fixed factors. We assessed homogeneity of variance by examining residual plots and the assumption of normality by examining Q–Q plots. If data did not meet the model assumptions, they were transformed. Because fish were genetically related (siblings), we also included brood as a random effect in all models of hormones, GSI and size. Averages are presented as mean ± SE. Results were considered significant at *α* < 0.05 (Thiese *et al.,* 2016).

### Hormones

In males, testosterone and the ratio of testosterone to oestradiol [an indicator of aromatase enzyme activity (Shang *et al.,* 2006, Friesen *et al.,* 2012)] were log transformed to improve model assumptions. We analysed both testosterone and the ratio of testosterone to oestradiol using LMMs with oxygen, turbidity, the interaction between oxygen and turbidity, and population as fixed factors; log-transformed SL as a covariate; and brood as a random effect. The time a sample was collected was initially included as a covariate in all hormone models, but it was not significant and subsequently removed from all models.

**Table 1:** Results for LMMs explaining the influence of population (swamp or river), oxygen (hypoxic or normoxic) and turbidity (clear or turbid) treatments and log-transformed SL on log-transformed testosterone and the log-transformed ratio of testosterone to oestradiol in F1 males

Variable	Effect	*df*	*T*	*P*
Testosterone	Oxygen	1,60.7	−0.809	0.422
*R*^2^_marginal_ = 0.297	Turbidity	1,62.8	−0.953	0.344
*R*^2^_conditional_ = 0.424	Population	1,14.1	0.796	0.439
	Oxygen × turbidity	1,59.0	−2.544	0.014^**^
	SL	1,70.6	1.677	0.098
Testosterone: oestradiol	Oxygen	1,61.1	−1.254	0.215
*R*^2^_marginal_ = 0.297	Turbidity	1,63.3	−1.322	0.191
*R*^2^_conditional_ = 0.410	Population	1,13.9	0.798	0.438
	Oxygen × turbidity	1,59.3	−1.965	0.054
	SL	1,68.0	−0.199	0.843

Brood was included as a random effect.

^**^Values are significant at *α* < 0.05.

### Body size

We analysed, log-transformed SL and log-transformed mass in males and females separately using LMMs with oxygen, turbidity and the interaction between oxygen and turbidity as fixed effects and brood as a random effect. Because sampling for size data was completed over approximately 8 months, age at the time of sampling was also included as a covariate in these models.

### Gonadosomatic index

To improve model assumptions, GSI was log transformed. We analysed GSI separately for males and females using LMMs with brood as a random effect and oxygen, turbidity, the interaction between oxygen and turbidity, and population as fixed factors and log-transformed SL as a covariate. For females, an additional fixed factor, brooding (yes/no), was included to account for females that were mouth brooding at the time of sampling, as their GSI was generally much lower (0.825 ± 0.124) than non-brooding females (3.516 ± 0.321).

### Fitness metrics

Finally, we analysed the effects of rearing treatment on egg number using a Poisson model (a Generalized Linear Model; GLM) with oxygen, turbidity, interaction between oxygen and turbidity as fixed factors, and the mass of the mother as a covariate. One brood was determined to be influential (Cook’s distance > 1) and was removed from all models. Additionally, the sample size of the hypoxic/turbid treatment combination produced by river females was low (*n* = 2), so the river and swamp populations were pooled for the analyses of egg number, batch weight and egg mass. We also analysed the batch weight of the broods (g) and egg mass (g; batch weight/# of eggs) using linear models with oxygen, turbidity, the interaction between oxygen and turbidity as fixed factors and the mass of the mother as a covariate.

## Results

### Male hormones

**Figure 1:**
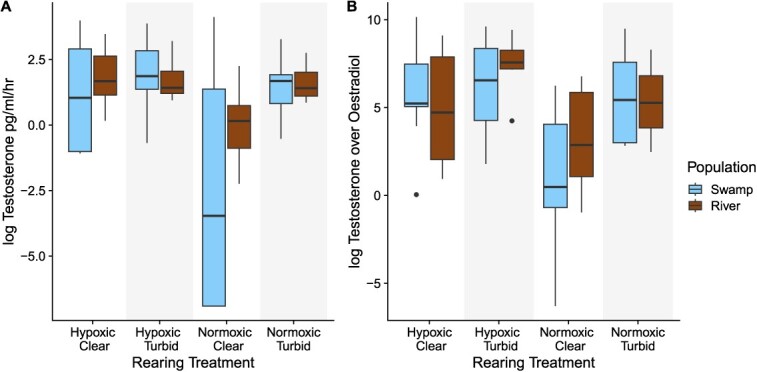
Boxplots of the median log-transformed (**A**) testosterone (pg/ml/h) and (**B**) the ratio of testosterone to oestradiol in males across four different treatment combinations (hypoxic or normoxic and turbid or clear) and two populations (swamp: blue; river: brown). The tails above and below the boxes represent, respectively, the maximum and minimum values of the sample; the dots represent outlier individuals.

We found that the excretion rate of testosterone in males was not affected by population or SL (Table 1). However, there was a significant interaction between oxygen and turbidity (Fig. 1A) where males not reared in turbid or hypoxic conditions had the lowest excretion rate of testosterone (Table 1). We also compared the ratio of testosterone to oestradiol in males as this is indicative of aromatase activity (Friesen *et al.,* 2012). Similar to testosterone, there was no effect of population or SL on the ratio of testosterone to oestradiol, but the interaction between oxygen and turbidity was not significant (*P* = 0.054).

### Body size

**Figure 2:**
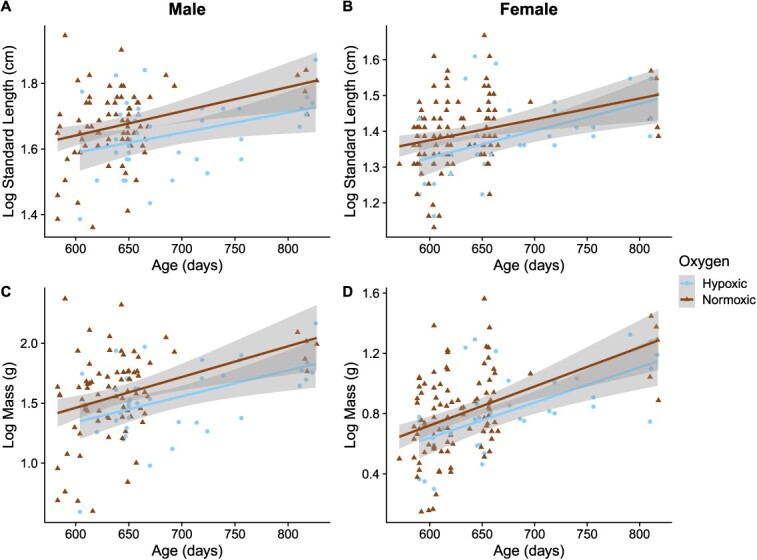
Linear relationship between (**A**) male log-transformed SL, (**B**) female log-transformed SL, (**C**) male log-transformed mass, and (**D**) female log-transformed mass and age across hypoxic (blue circles) and normoxic rearing treatments (brown triangles). Population and turbidity treatment were not significant, so data were pooled for clarity.

In males, SL and mass were affected similarly (Table 2 and Fig 2A and C). Males in the hypoxic treatment were significantly smaller (i.e. shorter mean SL, 5.2 ± 0.1 cm, and lower mean mass, 4.9 ± 0.2 g) than males in the normoxic treatment (mean SL, 5.4 ± 0.1 cm and mean mass, 5.1 ± 0.2 g), while older males, as expected, were larger (longer SL and higher mass). SL and mass did not differ between populations or turbidity treatments (Table 2). Additionally, the interaction between oxygen and turbidity was not significant.

**Table 2:** Results for LMMs explaining the influence of population (swamp or river), oxygen (hypoxic or normoxic), turbidity (clear or turbid) treatment and age at time of sampling on size data including log-transformed SL and log-transformed mass in males and females (brood was included as a random effect)

Variable	Effect	*df*	*T*	*P*
Male				
**SL**	Oxygen	1,109	2.537	0.013^**^
*R*^2^_marginal_ = 0.138	Turbidity	1,114	1.015	0.313
*R*^2^_conditional_ = 0.228	Population	1,14.9	0.276	0.786
	Age	1,111	3.836	<0.001^**^
	Oxygen ^*^ turbidity	1,107	−0.682	0.496
Mass	Oxygen	1,109	2.358	0.020^**^
*R*^2^_marginal_ = 0.116	Turbidity	1,115	1.023	0.308
*R*^2^_conditional_ = 0.248	Population	1,13.9	0.159	0.876
	Age	1,115	4.714	<0.001^**^
	Oxygen ^*^ turbidity	1,107	−0.957	0.341
Female				
**SL**	Oxygen	1,123	0.489	0.625
*R*^2^_marginal_ = 0.118	Turbidity	1,119	0.030	0.976
*R*^2^_conditional_ = 0.343	Population	1,17	−0.408	0.688
	Age	1,120	4.541	<0.001^**^
	Oxygen ^*^ turbidity	1,202	0.116	0.908
**Mass**	Oxygen	1,127	0.522	0.602
*R*^2^_marginal_ = 0.249	Turbidity	1,122	−0.445	0.657
*R*^2^_conditional_ = 0.382	Population	1,18.6	−0.245	0.809
	Age	1,123	6.774	<0.001^**^
	Oxygen ^*^ turbidity	1,124	0.792	0.430

^**^Values are significant at *α* < 0.05.

In females, SL did not differ between oxygen or turbidity treatments or populations (Table 2), though older females were larger (longer SL and higher mass). The mass of females was unaffected by oxygen, turbidity, population or their interaction (Table 2).

### Gonadosomatic index

**Figure 3:**
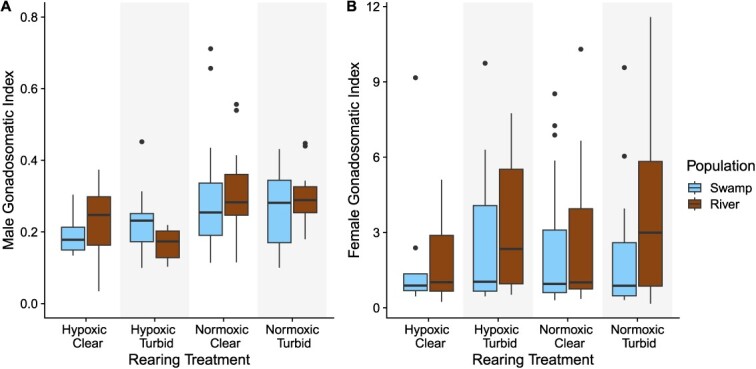
Boxplots of the median GSI in males (**A**) and females (**B**) across four different treatment combinations (hypoxic or normoxic and turbid or clear) and two populations (swamp: blue; river: brown). The tails above and below the boxes represent, respectively, the maximum and minimum values of the sample; the dots represent outlier individuals.

In males, GSI was generally low (0.261 ± 0.011). GSI of males did not differ between turbidity treatments. However, there was a significant effect of oxygen (Table 3). GSI of males was higher in the normoxic treatment for both populations and negatively correlated with SL (Fig. 3).

**Table 3:** Results for LMMs explaining the influence of population (swamp or river), oxygen (hypoxic or normoxic) and turbidity (clear or turbid) treatment, and log-transformed SL on log-transformed GSI in males and females [brood was included as a random effect for males and females; for females, whether a female was brooding (yes/no) at the time of sampling was also included as a fixed factor]

Variable	Effect	*df*	*t*	*P*
Male				
GSI	Oxygen	1,101	2.736	0.007^**^
*R*^2^_marginal_ = 0.090	Turbidity	1,101	−0.031	0.975
*R*^2^_conditional_ = 0.174	Population	1,101	0.191	0.849
	SL	1,101	−2.638	0.010^**^
	Oxygen × turbidity	1,101	0.472	0.638
Female				
**GSI**	Oxygen	1,113	0.450	0.654
*R*^2^_marginal_ = 0.497	Turbidity	1,117	−0.435	0.665
*R*^2^_conditional_ = 0.514	Population	1,11	1.669	0.122
	SL	1,115	1.629	0.106
	Brooding	1,110	−8.258	<0.001^**^
	Oxygen × turbidity	1,115	1.101	0.273

^**^Values are significant at *α* < 0.05.

In females, there was no effect of oxygen, turbidity or population on GSI (Table 3). However, many females were brooding at the time of sampling (45/127), and the mean ± SE for GSI of brooding females was lower (0.825 ± 0.124) than the GSI of females that were not brooding (3.516 ± 0.321; Table 3).

### Fitness metrics

**Figure 4:**
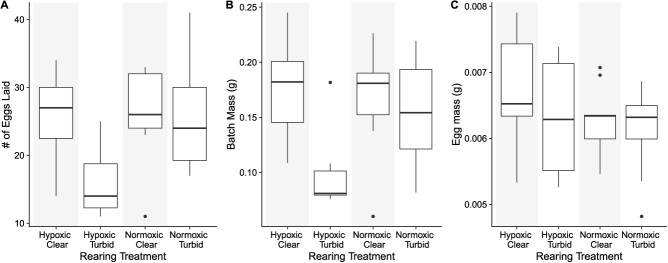
Boxplots of the median number of eggs laid (**A**), brood mass (g) (**B**) and egg mass (g) (**C**) of females from four different treatment combinations (hypoxic or normoxic and clear or turbid). We did not test for differences in population, so pooled data are presented. The tails above and below the boxes represent, respectively, the maximum and minimum values of the sample; the dots represent outlier individuals.

Over a period of 170 days, we collected 37 broods [one brood was an outlier (Cooke’s distance > 1) and excluded from these analyses]. Because the number of broods laid by females from the river population in the hypoxic/turbid treatment combination was low (*n* = 2), we did not test for differences between populations. We found that the interaction between oxygen and turbidity had a significant effect on the number of eggs laid by females; specifically, in the hypoxic/turbid treatment, females laid ~10 fewer eggs on average (Table 4 and Fig. 4A). Additionally, the overall mass of the brood was reduced in hypoxic and turbid treatments (Table 4 and Fig. 4B). However, the average mass of the individual eggs was not affected by the treatments or the mass of the mother (Table 4 and Fig. 4C).

## Discussion

Our study was designed to allow us to detect both plastic and population differences in response to hypoxia and turbidity by utilizing two populations that historically differ in their exposure to these stressors. In their natural environment, the river population is regularly exposed to elevated levels of turbidity, while the swamp population is exposed to chronic hypoxia. However, human activities are altering the environmental conditions in this region (Chapman *et al.,* 2008). For example, the river site described in this study was a wooded agricultural stream at the time of collection, but a road was built through the site in 2021. On a subsequent visit to the site in 2022, it was found to be deforested, dominated by papyrus and relatively high in turbidity and low in oxygen (point-in-time measurement, 13 July 2022; turbidity: 32.6 NTU; DO: 0.78 mg/l O_2_). *Pseudocrenilabrus multicolor* is still found at this site, so it would be useful for future studies to measure fitness at this modified site and compare it to other established low-oxygen sites. Importantly, in our laboratory study, we found that there was an interactive effect of the oxygen and turbidity rearing treatments on female reproductive success and male testosterone, even though *P. multicolor* regularly experiences hypoxia and turbidity in their natural environment.

### Stressor effects on sex hormones

While endocrine disruption due to hypoxia has been detected in females of this species and other fish, to our knowledge, this is the first study to measure the direct effects of turbidity on sex hormones. Our study provides evidence of endocrine disruption in male *P. multicolor* due to hypoxia. However, the effect of hypoxia on testosterone was dependent on the turbidity in which the fish was reared. Several other studies have found that hypoxia alters sex hormones in fish, including studies on female *P. multicolor* (Friesen *et al.,* 2012), Atlantic croaker (*Micropogonias undulatus*; Thomas and Rahman, 2012), Gulf killifish (*Fundulus grandis*; Landry *et al.,* 2007) and carp (*Cyprinus carpio*; Wu *et al.,* 2003). For example, Atlantic croakers from hypoxic sites exhibited decreased aromatase expression and a male-biased sex ratio (Thomas and Rahman, 2012). The increased ratio of testosterone to oestradiol could indicate inhibition of the aromatase enzyme, which requires oxygen to convert testosterone to oestradiol (Wu *et al.,* 2003). Additionally, hypoxia could have led to the downregulation in the expression of the aromatase enzyme. A study on female zebrafish (*Danio rerio*) found that a 14-day exposure to hypoxia led to the downregulation of aromatase expression in ovaries (Martinovic *et al.,* 2009). Such differences in aromatase expression could have pronounced physiological and behavioural outcomes. In the African cichlid, *Astatotilapia burtoni*, high aromatase expression was associated with dominance, and experimentally inhibiting the aromatase enzyme decreased aggression (Huffman *et al.,* 2013). While we did not measure behaviour and hormones concurrently, we found in a separate study using the same fish that males reared in hypoxic environments engaged in more competitive and courtship behaviours than those raised in normoxic environments (Williams *et al.,* 2024). Studies that directly test the effects of aromatase inhibition on the physiology and behaviour of fish would be useful to disentangle the energetic costs from endocrine disruption caused by hypoxia.

**Table 4:** GLMs and LMMs explaining the effects of oxygen and turbidity on fitness variables [number of eggs, brood mass (g) and egg mass (g); brood mass/number of eggs]

Variable	Effect	*df*	*T*	*P*
Number of eggs laid	Oxygen	1,31	3.891	<0.001^**^
	Turbidity	1,31	4.011	<0.001^**^
	Oxygen × turbidity	1,31	−3.042	0.002^**^
	Mother’s mass	1,31	−0.082	0.935
Brood mass	Oxygen	1,31	2.351	0.025^**^
	Turbidity	1,31	3.060	0.005^**^
	Oxygen × turbidity	1,31	−2.058	0.048^**^
	Mother’s mass	1,31	0.205	0.839
Egg mass	Oxygen	1,31	−0.560	0.579
	Turbidity	1,31	0.960	0.345
	Oxygen × turbidity	1,31	−0.678	0.503
	Mother’s mass	1,31	0.982	0.334

^**^Values are significant at *α* < 0.05.

While there are several examples of hypoxia affecting hormone levels in fish, this is the first study, to our knowledge, that documents differences in reproductive hormones associated with turbidity. Because increased male–male aggression can induce surges in testosterone (Wingfield *et al.,* 1990), turbidity could affect sex hormones indirectly by either increasing or decreasing aggression through alterations to the sensory (e.g. visual) environment. In a separate study using the same fish, we found that males reared in turbid environments engaged in fewer competitive and courtship behaviours than those raised in clear environments (Williams *et al.,* 2024). Turbidity can also hormonally impact fish by elevating stress hormones (Kemp *et al.,* 2011; Gray, 2016), which can interfere with the production of sex hormones (Wingfield and Sapolsky, 2003). For instance, in Japanese flounder (*Paralichthys olivaceus*), the administration of cortisol induced female-to-male sex reversal through inhibition of aromatase expression (Yamaguchi *et al.,* 2010). The physiological mechanisms through which turbidity could affect hormone levels requires further investigation. However, there are numerous examples of turbidity influencing behaviour in both positive directions [e.g. guppies *P. reticulata* (Ehlman *et al.,* 2015) and Atlantic cod *Gadus morhua* (Meager and Batty, 2007)] and negative directions [e.g. Lake Malawi cichlids (Gray *et al.,* 2011), walleye *Sander vitreus* (Nieman and Gray, 2018) and emerald shiner *Notropis atherinoides* (Nieman and Gray, 2018)]. Turbidity is an increasingly detrimental aquatic stressor (Gray, 2016). Therefore, further research is needed to determine the mechanisms that produce changes in sex hormone concentrations (e.g. by changes to the visual environment or elevation of stress hormones) under turbid conditions and their potential consequences.

### Stressor effects on investment in reproductive tissues

Hypoxia negatively affected the investment of males into reproductive tissues, quantified as GSI. Other studies in fishes have also found that hypoxia leads to lower testicular growth, including studies on Atlantic croakers (Thomas *et al.,* 2007; Thomas and Rahman, 2012), Neumayer’s barb (*Barbus neumayeri*; Martínez *et al.,* 2015) and carp (*C. carpio*; Wu *et al.,* 2003). While we found that hypoxia affected male investment in reproductive tissues, we did not find an effect of turbidity on GSI in males. This contradicts the results of a previous rearing study on *P. multicolor* that found that males in turbid environments had a higher GSI (Atkinson, 2019). However, the males in that experiment were fed a low-carotenoid diet, which could have affected their energy allocation. In females, we also did not find an effect of hypoxia or turbidity on GSI. However, due to a small sample size of non-brooding females, comparisons across treatments and populations are challenging (i.e. brooding females had much smaller ovaries because fully developed ova had been recently released). In contrast to a past study, we did not find any differences in egg number due to rearing under hypoxia alone (Reardon and Chapman, 2009). While we did not measure egg size directly in this study, *P. multicolor* from hypoxic sites tends to lay larger batches of smaller eggs (Reardon and Chapman, 2009). Small eggs have a larger surface area-to-volume ratio, which could increase oxygen uptake (Reardon and Chapman, 2009). Additionally, small eggs tend to produce small juveniles (Reardon and Chapman, 2009), and we found that males reared under hypoxic conditions were smaller than those reared under normoxic conditions. Importantly, we found that the number of eggs and overall batch weight laid by females were reduced in the hypoxic/turbid combination (a novel combination of stressors for these fish), suggesting that increasing the number of stressors can negatively affect fitness, even when organisms are well adapted to the stressors individually.

### Hypoxia effects on size

Smaller size is assumed to be a cost of hypoxia that can also reduce fitness (Chapman, 2021), as increased size is often associated with greater fecundity, greater survival and greater mating success in various plants, invertebrates and vertebrates (Kingsolver and Pfennig, 2004; Kingsolver and Pfennig, 2007). Other studies have found that hypoxia reduces growth and increases the chance of malformations (Pollock *et al.,* 2007). Offspring of *P. multicolor* from swamp sites tend to be shorter, have a lower mass and have a shorter brooding period (Reardon and Chapman, 2012). Similarly, we found that adult males reared under hypoxic conditions were smaller (shorter SL and lower mass) than males reared under normoxic conditions in both populations. This suggests that both populations retain plasticity of size in response to the DO rearing environment. As this species is highly hypoxia-tolerant, small size could be an adaptation to hypoxia, as it relates to smaller embryo size (i.e. increased volume to surface ratio) and shorter brooding intervals (i.e. potential for decreased cost of brooding for mother). Conversely, female size was unaffected by hypoxia, so the reduced egg count under hypoxic/turbid conditions was not due to a reduction in female size. Smaller size could reduce hypoxia tolerance during severe hypoxia that requires anaerobic metabolism, as larger fish have greater glycogen storage and lower mass-specific metabolic rates (Nilsson and Östlund-Nilsson, 2008). However, smaller fish may still prefer hypoxic sites even if they are less hypoxia-tolerant compared to larger fish. For example, small oscars, *Astronotus ocellatus,* which are less hypoxia-tolerant, prefer hypoxic areas as refuges from predation (Sloman *et al.,* 2006). Overall, our research suggests that even in populations that are accustomed to hypoxic conditions, chronic hypoxia leads to smaller fish compared to siblings reared in normoxic conditions.

## Concluding Remarks

Several of the trait outcomes due to hypoxia and/or turbidity could be interpreted as costs of surviving in hypoxic and turbid conditions. However, *P. multicolor* is well adapted to these stressors and could mitigate or compensate for changes in hormone levels or morphological traits. *Pseudocrenilabrus multicolor* is a species that has adapted to reproducing in chronic hypoxia, and the benefits of living in hypoxic swamps (e.g. refuge from parasites and predators; Chapman *et al.,* 2002; Chapman, 2021) could be important to offset such costs. However, the reduction in the fitness of females reared in the hypoxic/turbid treatment suggests that even species accustomed to surviving in certain environmental conditions could be negatively affected when faced with multiple stressors simultaneously. This research contributes to our understanding of how multiple stressors could influence fitness in aquatic organisms. Multiple stressors are a growing conservation issue for managers (Côte *et al.,* 2016). Understanding how multiple stressors will affect organisms is inherently difficult, as responses are likely to differ based on the number of stressors, type of stressors, timing and duration of exposure, species and life stage (Côte *et al.,* 2016; Kaunisto *et al.,* 2016; Jackson *et al.,* 2021). However, our research suggests that an increasing number of stressors could have negative outcomes for reproductive success. Other human-induced environmental changes (e.g., warming, invasive species, pollution; Chapman *et al.,* 2008) in the Lake Victoria region could also be interacting to affect reproduction. The combined effects of these stressors on fish reproduction remain to be determined. Such research will be vital to understand how multiple stressors affect reproductive success as these stressors increase due to human activities in aquatic habitats.

## Supplementary Material

Web_Material_coae066

## Data Availability

All data are available on Dryad: https://doi.org/10.5061/dryad.k0p2ngfdg.
