# Supplementary material for: Multiple stressors disrupt sex hormones and fitness outcomes: effects of hypoxia and turbidity on an African cichlid fish
Source: Conserv Physiol. 2024 Oct 22;12(1):coae066. doi: 10.1093/conphys/coae066 (PMC11496714; doi:10.1093/conphys/coae066)
Supplement: Web_Material_coae066 [file web_material_coae066.docx]

Supplementary Table 1: Sample size of males or females used in each analysis.

|  | Swamp (Lwamunda) | | | | River (Ndyabusole) | | | |
| --- | --- | --- | --- | --- | --- | --- | --- | --- |
|  | Normoxic, Clear | Normoxic, Turbid | Hypoxic, Clear | Hypoxic, Turbid | Normoxic, Clear | Normoxic, Turbid | Hypoxic, Clear | Hypoxic, Turbid |
| Testosterone ♂ | 10 | 10 | 10 | 10 | 10 | 10 | 9 | 8 |
| Estradiol ♂ | 10 | 10 | 10 | 10 | 10 | 10 | 9 | 8 |
| Testosterone: estradiol ♂ | 10 | 10 | 10 | 10 | 10 | 10 | 9 | 8 |
| Standard Length ♂ | 16 | 20 | 9 | 11 | 23 | 20 | 11 | 8 |
| Standard Length ♀ | 30 | 25 | 8 | 15 | 21 | 23 | 7 | 8 |
| Mass ♂ | 16 | 20 | 9 | 11 | 23 | 20 | 11 | 8 |
| Mass ♀ | 30 | 25 | 8 | 15 | 21 | 23 | 7 | 8 |
| GSI ♂ | 16 | 17 | 9 | 11 | 21 | 14 | 11 | 8 |
| GSI ♀ | 28 | 24 | 8 | 14 | 18 | 20 | 7 | 8 |
| Egg Number ♀ | 4 | 5 | 5 | 4 | 5 | 5 | 6 | 2 |
| Batch Weight ♀ | 4 | 5 | 5 | 4 | 5 | 5 | 6 | 2 |
| Egg Mass ♀ | 4 | 5 | 5 | 4 | 5 | 5 | 6 | 2 |

*Supplementary Information*

Estradiol measurements in males were generally low, and they were undetectable in 38 of the 77 fish measured. The undetectable measurements were spread relatively evenly across populations (swamp 52.5% undetectable and river, 45.9% undetectable). However, across treatment groups, the two hypoxic treatments appeared to have more undetectable measurements than the normoxic treatment combinations (hypoxic/clear 52.6% undetectable, hypoxic/turbid 72.2% undetectable compared to normoxic/clear 35.0% undetectable, and normoxic/turbid 40.0% undetectable).


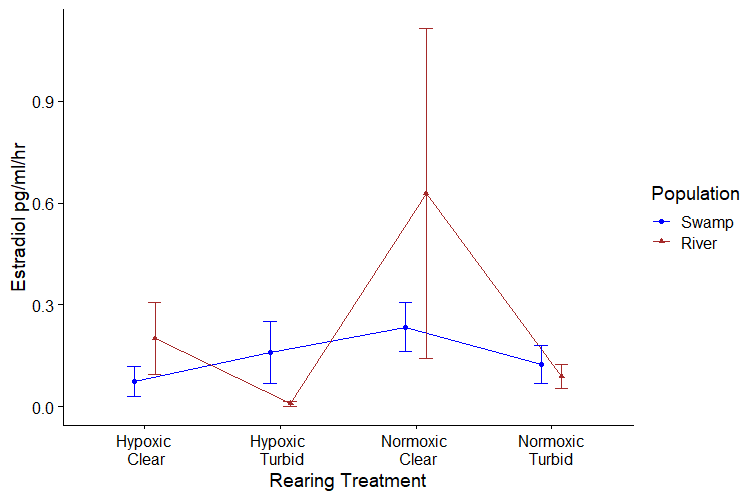


Supplementary Figure 1: Mean ± SE Estradiol (pg/ml/hr) of females from four different treatment combinations (hypoxic or normoxic and clear or turbid) and two populations (blue circles = swamp, brown triangles = river).
